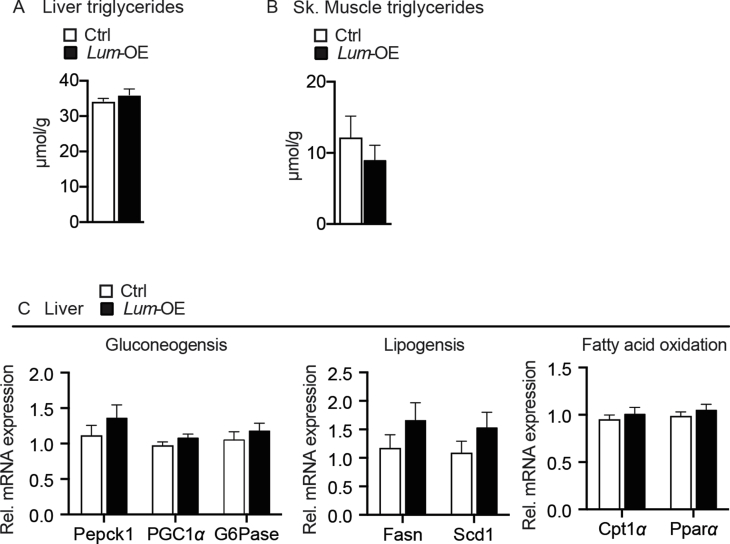# Diet-dependent function of the extracellular matrix proteoglycan Lumican in obesity and glucose homeostasis

**DOI:** 10.1016/j.molmet.2018.10.007

**Published:** 2018-10-23

**Authors:** G. Wolff, A.E. Taranko, I. Meln, J. Weinmann, T. Sijmonsma, S. Lerch, D. Heide, A.T. Billeter, D. Tews, D. Krunic, P. Fischer-Posovszky, B.P. Müller-Stich, S. Herzig, D. Grimm, M. Heikenwälder, W.W. Kao, A. Vegiopoulos

**Affiliations:** 1 DKFZ Junior Group Metabolism and Stem Cell Plasticity, German Cancer Research Center, Heidelberg, Germany; 2 Heidelberg University Hospital, Dept. of Infectious Diseases/Virology, BioQuant Center, Heidelberg, Germany; 3 Division Chronic Inflammation and Cancer, German Cancer Research Center (DKFZ), Heidelberg, Germany; 4 Department of General, Visceral, and Transplantation Surgery, University of Heidelberg, Heidelberg, Germany; 5 Division of Pediatric Endocrinology and Diabetes, Department of Pediatrics and Adolescent Medicine, University Medical Center, Ulm, Germany; 6 Light Microscopy Facility, German Cancer Research Center (DKFZ), Heidelberg, Germany; 7 Helmholtz Center Munich, Institute for Diabetes and Cancer IDC, Neuherberg, Germany; 8 Joint Heidelberg-IDC Translational Diabetes Program, Heidelberg University Hospital, Heidelberg, Germany; 9 German Center for Infection Research, Partner Site Heidelberg, Germany; 10 Department of Ophthalmology, University of Cincinnati, Cincinnati, OH, USA

**Keywords:** Lumican, ECM, Inflammation, Obesity, Adipose, Insulin

## Abstract

**Objective:**

Extracellular matrix remodeling is required for adipose expansion under increased caloric intake. In turn, inhibited expandability due to aberrant collagen deposition promotes insulin resistance and progression towards the metabolic syndrome. An emerging role for the small leucine-rich proteoglycan Lumican in metabolically driven nonalcoholic fatty liver disease sparks an interest in further understanding its role in diet-induced obesity and metabolic complications.

**Methods:**

Whole body ablation of Lumican (*Lum*^*−/−*^) gene and adeno-associated virus-mediated over-expression were used in combination with control or high fat diet to assess energy balance, glucose homeostasis as well as adipose tissue health and remodeling.

**Results:**

Lumican was found to be particularly enriched in the stromal cells isolated from murine gonadal white adipose tissue. Likewise murine and human visceral fat showed a robust increase in Lumican as compared to fat from the subcutaneous depot. Lumican null female mice exhibited moderately increased fat mass, decreased insulin sensitivity and increased liver triglycerides in a diet-dependent manner. These changes coincided with inflammation in adipose tissue and no overt effects in adipose expandability, i.e. adipocyte formation and hypertrophy. Lumican over-expression in visceral fat and liver resulted in improved insulin sensitivity and glucose clearance.

**Conclusions:**

These data indicate that Lumican may represent a functional link between the extracellular matrix, glucose homeostasis, and features of the metabolic syndrome.

## Highlights

• The extracellular matrix proteoglycan Lumican (Lum) is particularly enriched in stromal cells within white adipose tissue.
• Visceral fat from obese patients displays increased levels of Lum compared to subcutaneous fat.
• *Lum*-Ko female mice exhibit decreased insulin sensitivity and increased triglycerides upon high-fat diet (HFD) feeding.
• *Lum*-Ko female mice on HFD have increased inflammation in white fat in the absence of overt effects on adipocyte formation.
• · Lum over-expression in visceral fat and liver resulted in improved insulin sensitivity and glucose clearance.

## 1 Introduction

Adipose tissue expansion in the face of caloric excess initiates concomitant processes including angiogenesis, inflammation, and extracellular matrix (ECM) remodeling [1], [2]. Persistent adipose growth promotes obesity, progression towards the metabolic syndrome, and type-2 diabetes. The regulation of insulin sensitivity as a pathogenic factor in the metabolic syndrome represents an important target driving current and future therapeutic treatments [1], [3], [4]. The “expandability hypothesis” suggests that fat accumulation in organs outside of the adipose tissue leads to cell death, inflammation, and insulin resistance providing one explanation for obesity-driven insulin resistance as well as insulin-sensitive obesity [4], [5], [6].

Remodeling the ECM in adipose tissue requires reciprocal processes of depositing new matrix proteins, mainly collagens, and breaking down via proteases such as matrix metalloproteinases (MMPs) [4]. Moreover, altered collagen expression can directly improve metabolic health as reported for body mass, insulin sensitivity, and liver triglycerides in collagen VI-null *ob/ob* obese mice [7]. The small leucine-rich proteoglycan Lumican (Lum) binds collagen and is associated with repair processes in collagen-rich connective tissues [8], [9], [10]. Lum has been shown to bind directly to MMPs and integrins, to decrease MMP activity and slow tumor progression [11], [12]. Lum also interacts with inflammatory cells via integrins and promotes fibrocyte differentiation when stimulated by tumor necrosis factor alpha (TNFα) [13], [14]. Recent reports have implicated Lum as a marker for fibrotic progression in nonalcoholic fatty liver disease (NAFLD) and nonalcoholic steatohepatitis (NASH) [15], [16], [17]. In patients with NAFLD and NASH the hepatic collagen fractional synthesis rate, which is an established correlate of fibrotic disease progression, correlated with increasing plasma Lum levels suggesting Lum could be developed as a diagnostic tool to measure liver fibrosis and NAFLD/NASH staging [16].

Although Lum has been connected to fibrosis [18], [19], immune function [13], [14], [20] or cancer progression [11], and has even been implicated as biomarker in obesity-driven liver disease [21], its roles in obesity development and the metabolic syndrome have not been explored. Preliminary results from microarray analysis revealed that Lum mRNA was differentially expressed in white adipose tissue of mice fed high-fat diet (HFD) (unpublished results). We decided to explore the impact of Lum loss (*Lum-*Ko, total body null mice) and Lum over-expression (OE) on metabolism and adipose tissue quantity and quality. In this study, we exposed male and female mice to HFD and observed a sex-specific effect on fat accumulation in female *Lum-*Ko mice that coincided with increased inflammation and insulin resistance. Likewise, *Lum-*OE in the visceral fat and liver improved glucose clearance and insulin sensitivity. Together these results suggest a role of Lum in systemic glucose homeostasis and the development of metabolic complications associated with obesity.

## 2 Materials and methods

### 2.1 Animals and experimental models

Lumican null (*Lum-*Ko) mice originated from the Kao Lab at the University of Cincinnati, (Cincinnati, OH, USA) [22] and C57BL/6 mice were purchased from Charles River Laboratories (Sulzfeld, Germany) at 4–5 weeks of age. Mice were housed at room temperature with 12-h light-dark cycle on control diet (Research diets, New Brunswick, NJ, USA, D12450j) or high-fat diet (60% kcal from fat, Research diets, D12492) for 3, 8, or 10 weeks as indicated. Mice were housed in sex-matched groups of 2–3 animals with *ad libitum* access to food and water. EchoMRI was used to measure body composition (Echo Medical Systems, Houston, TX, USA). At the conclusion of the study, tissues were flash frozen for analysis or placed in histofix (formaldehyde, Carl Roth GmbH, Karlsruhe, Germany). All experiments were performed in accordance with the European Union directives and the German animal welfare act (Tierschutzgesetz) and were approved by local authorities (Regierungspräsidium Karlsruhe).

### 2.2 TSE PhenoMaster

Oxygen consumption (indirect calorimetry), food intake, and locomotor activity measurements were performed for individually housed mice at 22 °C in the PhenoMaster Home Cage System (TSE system, Bad Homburg, Germany). Total energy expenditure (TEE) was calculated using the Weir equation where metabolic rate (kcal/day) = 1.44 (3.94 × VO_2_ + 1.11 × VCO_2_) [23].

### 2.3 Serum insulin, liver triglycerides, HOMA-IR, GTT/ITT

Serum insulin levels were determined by ELISA Kit (80-INSMS-E01-AL, ALPCO, Salem, NH, USA) according to the manufacturer's protocol and the Mithras Microplate Reader (Berthold Technologies GmbH & Co, Bad Wildbad, Germany). Concentration was calculated based on standard dilution series using Elisa analysis web-resource (http://www.elisaanalysis.com/). Blood glucose was determined with an Accu-Check glucometer (Roche Diagnostics, Mannheim, Germany). HOMA-IR was calculated using the formula: HOMA-IR (mmol l-1 × μU ml-1) = fasting blood glucose (mmol l-1) × fasting serum insulin (μU ml-1)/22.5. Liver and muscle triglycerides were extracted as previously described [24] and determined with the Serum Triglyceride Determination Kit (TR0100, Sigma Aldrich, Munich Germany) and the Mithras Microplate Reader (Berthold Technologies GmbH & Co, Germany). Insulin tolerance test (ITT) and glucose tolerance test (GTT) were performed as previously described [25].

### 2.4 Immunofluorescence/Immunohistochemistry

gWAT and iWAT were fixed with 4% Histofix (Carl Roth) at room temperature for 24 h then embedded in paraffin. Blocks were cut on a RM2245 microtome (Leica, Nussloch, Germany) into sections 5-μm thick sections each and placed on glass slides. Sections were de-paraffinized with xylene and ethanol dilutions to rehydrate. For Caveolin-1 staining, slides were subjected to antigen retrieval in citrate buffer (pH 6.0) by boiling at 95 °C for 20 min. The slides were blocked with 2% bovine serum albumin (BSA) in PBS for 1 h at room temperature followed by overnight incubation at 4 °C with rabbit α-Caveolin-1 polyclonal antibody (Cell Signaling, Danvers, MA, USA), diluted 1:400 in 2% BSA in PBS (Cell Signaling). Following washes with PBS, goat α-rabbit IgG-Alexa Fluor® 488 secondary antibody at 1:400 dilution in 2% BSA/PBS (Thermo Fisher Scientific, Rockford, IL, USA) was applied for 60 min at room temperature. Slides were washed, and mounted with ProLong® Gold Antifade Reagent (Cell Signaling). Images were acquired using the Zeiss Cell Observer with identical settings and acquisition times for all samples and analyzed with Fiji software (ImageJ, https://imagej.nih.gov/ij/). In brief, images were background-subtracted using Rolling ball algorithm, median filtered, skeletonized. Upon application of the Analyze Particles tools, the cell selections were added to the region of interest (ROI) manager with areas determined [26]. Adipocyte diameter and volume were estimated based on area using following formulas: diameter = 2√ (mean cell area/π) μm and volume = π (diameter^3^/6) μm^3^. Number of adipocytes per gram of adipose tissue was calculated as follows: 1) conversion of the volume from μm^3^ to a volume in picolitres (pl) (1 pl = 1000 μm^3^), 2) calculation of the mass of a cell, where mass = volume × density and the density of adipose tissue can be assumed to be 0.96 g ml^−1^, mean cell mass = mean cell volume × 0.96, 3) calculation of the number of cells per mg of tissue as number of cells per mg tissue = 1/mean cell mass. The absolute number of adipocytes per depot was estimated as = number of adipocytes per gram × gram mass of depot.

For F4/80 and Cd11c staining slides were processed as previously reported [27] using the BondMax (Leica Biosystems, Wetzlar, Germany), rat α-F4/80 antibody (diluted 1:120, Linaris Biologische Produkte GmbH, Dossenheim, Germany, #13422.00500), hamster α-Cd11c (diluted 1:5000, BD Bioscience, Heidelberg, Germany, #553799) and HRP-conjugated 2° antibodies (1:1000) with hematoxylin counterstaining. Images were acquired using the Zeiss Axioplan microscope with identical settings and acquisition times for all samples. F4/80-positive cells and crown like structures (CLS) were averaged from 10 independent images sections, counted blindly. Pikro-Sirius red staining was performed on a Leica staining machine (model #ST5020). Following rehydration and rinsing, slides were stained with Pikro-Sirius red (Morphisto GmbH, Vienna, Austria) for 60 min and dehydrated. Images were acquired using the Upright Zeiss Axiophot microscope with identical settings and acquisition times for all samples.

### 2.5 Human subjects

Biopsies from human abdominal subcutaneous fat and visceral omental fat were collected during bariatric surgery at the Department of Surgery, University Hospital Heidelberg, with approval by the Institutional Review Board of the Medical Faculty of the University of Heidelberg in accordance with the declaration of Helsinki and its later amendments. Preoperative informed consent was obtained from all patients for the use of samples. Biopsies were snap-frozen upon collection. Average patient age was 49 years, BMI>30, n = 11 females and n = 10 males were evaluated in this study.

For cell isolation, mammary adipose tissue was obtained from n = 5 women undergoing plastic surgery (mean BMI 31.2 kg/m^2^, range 24.8–36.0; mean age 46.6 years; range 20–65). The study was approved by the University Ulm ethical committee (vote no. 300/16) and all patients gave written informed consent. Cells were isolated by collagenase digestions as described [28]. Adipocytes and stromal-vascular cells (SVC) were separated by centrifugation.

### 2.6 RNA isolation and qRT-PCR analysis

RNA was extracted from snap-frozen tissues using QIAZOL (QIAGEN, Hilden, Germany) and the RNeasy micro kit (QIAGEN) including DNase treatment or the Direct-zol RNA Miniprep kit (human mammary fat, Zymo Research, Freiburg, Germany) following the manufacturer's protocol. Complementary DNA (cDNA) synthesis was performed RNA using QuantiTect_ Reverse Transcription Kit (Qiagen). Quantitative real-time polymerase chain reaction (qRT**–**PCR) was performed using TaqMan Gene Expression Assays (Life Technologies, Darmstadt, Germany) and TaqMan Gene Expression Master Mix on a StepOnePlusTM Real-Time PCR System (Life Technologies). Analysis of mammary fat-derived RNA was performed with the Sso Advanced Universal SYBR Green Supermix (BioRad, München, Germany) on a CFX Connect qPRC cycler (BioRad) with custom primers for LUM (forward-TAACTGCCCTGAAAGCTACCC, reverse GGAGGCACCATTGGTACACTT) and TF2B (forward-TGGGATCTGAATGGCGAACTTTCAGCAATGAC, reverse TCCTGTGCCCTT GCCAATCATGGTAGAC). Relative expression was calculated using the ΔΔCT method with normalization to Tbp/TBP (TATA box binding protein), 18S rRNA (pancreas) or TF2B (mammary fat).

### 2.7 Western blot

Proteins were extracted with RIPA buffer with protease and phosphatase inhibitors (Sigma–Aldrich), separated by SDS-PAGE (10%) and blotted onto nitrocellulose membranes (0.45 μM, BioRad, USA) with a BioRad wet blot tank system. The blots were subjected to blocking with 5% BSA and overnight incubation at 4 °C with rabbit α-Lumican (1:1000, R & D Systems Minneapolis, MN, USA, #AF2745), rat α-Cd11c (1:1000, R & D Systems, #MAB11241) or mouse α-Vcp (1:5000, clone 5, cat. no. ab11433, Abcam, UK), followed by HRP-conjugated 2° antibodies, ECL Select Western Blot detection reagent (GE Healthcare Life Sciences, USA) and imaging using ChemiDoc XRS+ (BioRad, USA).

### 2.8 Isolation and differentiation of adipose progenitor cells

To isolate progenitor cells for seeding or sorting [25], gWAT was dissected and digested as described previously [29], [30]. The Lin-Sca1+ fraction cells were counted using a hemocytometer and seeded on laminin-coated 24-well plates. Cells were seeded at 30,000 cells/well in DMEM supplemented with 100 U/ml penicillin-streptomycin (Thermo Fisher Scientific), 10% (v/v) fetal calf serum (FCS, Life Technologies) and 10 ng/ml recombinant basic fibroblast growth factor (bFGF, R&D Systems). At approximately 90% confluence, cells were treated with DMEM, 10% FCS, 1% penicillin/streptomycin, 1 μg/ml insulin, 500 nM dexamethasone, 3 nM triiodothyronine (T3) (Sigma–Aldrich) for 2 days, followed by DMEM. 5% FCS, 1 μg/ml insulin and 3 nM T3 for up to 6 additional days. For Oil red O staining cells were washed with PBS and 4% formalin was applied for 5 min. Following washing with 60% isopropanol, cells were dried and filtered Oil red O (8.5 mM stock) 60% solution with water was added to each well. After incubation and washing, images were taken with a Zeiss AxioVert.A1 microscope (camera Cam/CM1, grayscale). Oil red O was eluted with 100% isopropanol and measured at 490 nm with a Mithras Microplate Reader (Berthold Technologies GmbH & Co, Germany). To account for systematic differences between experiments, the mean O.D. value for each sample was scaled to the mean of all samples in each experiment.

### 2.9 Adeno-associated virus vectors

pSSV9-CAG-Lum (AAV-Lum) was cloned by insertion of an EcoRI-NotI fragment from pCMV6-Lum (Origene/Biocat, Heidelberg, Germany, #MC207318-OR) containing the complete mouse *Lum* coding sequence, into the pSSV9 backbone [31] containing the CMV enhancer/chicken beta-actin promoter (CAG) element. The control vector AAV-GFP contained the GFP coding sequence in place of the *Lum* sequence. For the production of recombinant AAVs, HEK293T cells were triple-transfected with 1) the pSSV9 vector, 2) the p5E18-VD2/8mut6 helper plasmid containing AAV2 *rep* and AAV8 mutant region 6 *cap* genes [32], as well as 3) the pDGΔVP adenoviral helper plasmid using polyethylenimine as transfection reagent. The cells were collected by scraping and centrifugation, lysed by freeze/thaw and treated with Benzonase AAV particles were purified by an iodixanol density gradient as described previously [33]. The AAV vector-containing iodixanol fraction was dialyzed and concentrated with an Amicon Ultra-15 tube.

### 2.10 Statistical analysis

Data were graphed and analyzed using GraphPad Prism 6 software (La Jolla, CA, USA) and SigmaPlot 12.5 (Systat Software GmbH, Erkrath, Germany). mRNA expression, tissue mass data (within tissue), and metabolic measures (area under the curve, insulin, HOMA-IR) for *Lum-*Ko experiments were analyzed by 2-way ANOVA with posthoc Tukey's test. Expression data were log-transformed before analyzing in order to approximate a normal distribution. Body mass data, ITT, and GTT were analyzed using 2-way ANOVA with repeated measures (RM) followed by posthoc Sidak test. Correlations were evaluated using Pearson's correlation coefficient. Analysis of covariance (ANCOVA) was performed for TSE data followed by 2-way ANOVA with posthoc Tukey's test. For overexpression experiments, unpaired *t*-test was performed in all cases except for insulin and HOMA-IR comparison at 0min and 30min post glucose administration, where paired *t*-tests were used. All statistical tests were applied as indicated, and p < 0.05 was considered significant. Data are plotted as mean ± S.E.M.

## 3 Results

### 3.1 High fat diet increases Lum mRNA levels in gonadal fat

**Figure 1:**
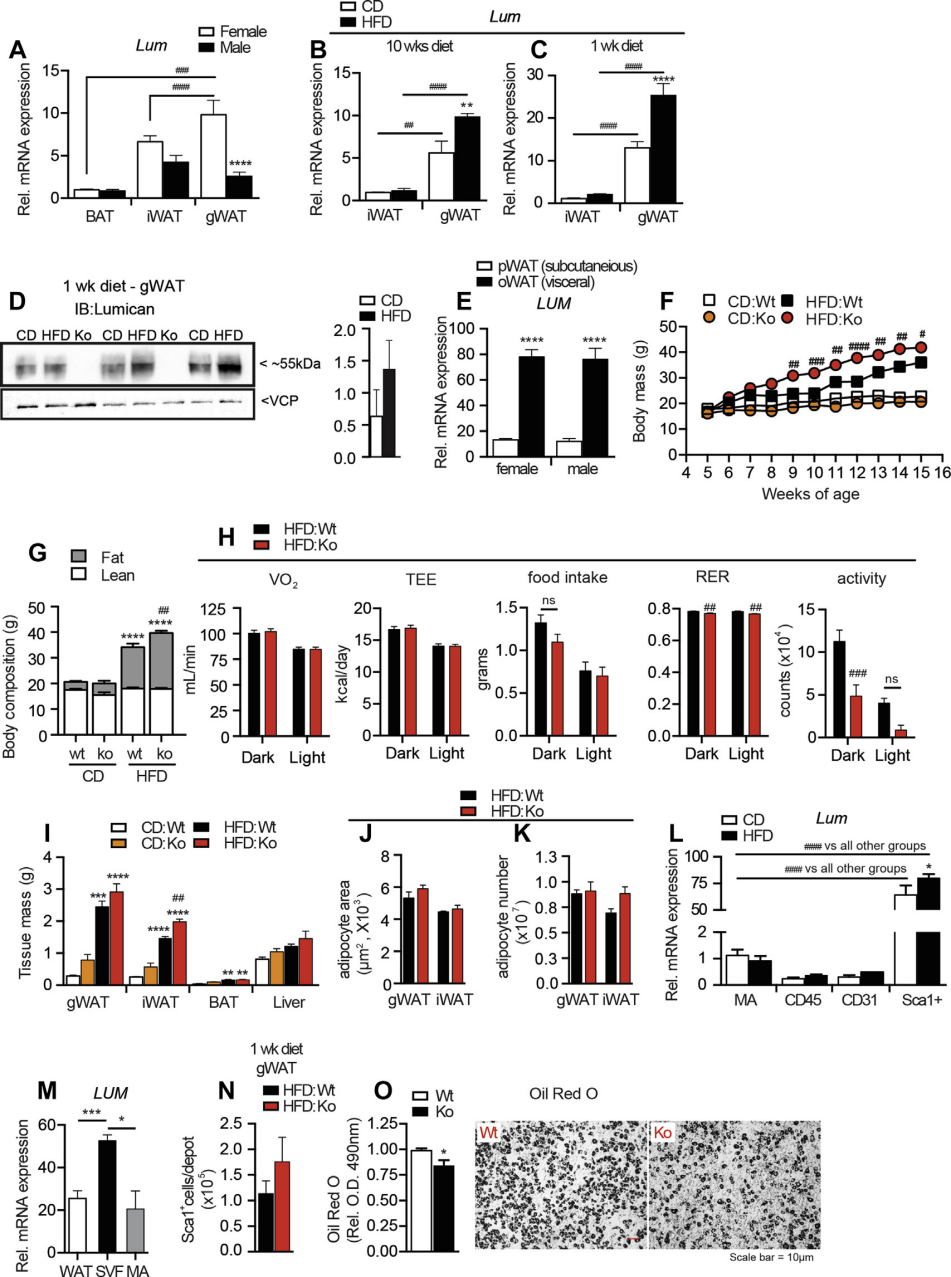
***Lum-*Ko females have increased fat mass on HFD**. *Lum* mRNA expression across tissue and sex on CD (A). *Lum* mRNA expression in iWAT and gWAT female mice on diet for 10 weeks (B) or 1 week (C), protein (D). Human samples (male and female) *LUM* mRNA expression in pWAT and oWAT (E). Body mass for *Lum-*Ko and Wt female mice (F). EchoMRI at 15 weeks of age (G). TSE Phenomaster “CaloCages” data acquired for 1 week (H). Tissue masses at 15 weeks of age (I). iWAT and gWAT mean adipocyte areas (J) and adipocyte number per depot (K). *Lum* mRNA expression from gWAT sorted adipose populations (L). *LUM* RNA from mammary adipose tissue (WAT), isolated adipocytes (MA) as well as isolated stromal-vascular cells (SVF) (M). Number of Sca1^+^ cells from *Lum-*Ko and Wt female mice on 1 week of diet (N). Differentiated progenitor cells stained for Oil Red O, quantification and a representative image (O). Data are mean ± SEM. (A) n = 6, (B) n = 3–5, (C–D) n = 9–10, (E) n = 9–11, (F to K) n = 3–8, (L) n = 3, (M) n = 5, (N–O) n = 3–4 per group. Statistical analysis was performed using two-way ANOVA with posthoc Tukey's test for all panels except F and M-O. For F, two-way ANOVA with RM and posthoc Sidak test was used. For M one-way ANOVA with posthoc Tukey's, for N and O, t-Test; *p < 0.05, **p < 0.01, ***p < 0.001, ****p < 0.0001 vs. CD:Wt. (#p < 0.05, ##p < 0.01, ###p < 0.001, ####p < 0.0001 HFD:Ko vs. HFD:Wt, unless otherwise indicated). pWAT = parietal white adipose tissue, oWAT = omental white adipose tissue, BAT = brown adipose tissue, iWAT = inguinal white adipose tissue, gWAT = gonadal white adipose tissue; hematopoietic (CD45), endothelial (CD31) or mature adipocytes (MA).

To investigate the contribution of proteins involved in extracellular matrix remodeling within adipose tissue during diet-induced obesity, we explored cohorts of male and female mice under control diet (CD) or HFD. Female mice maintained on CD for 8 weeks had significantly higher Lum mRNA levels in gonadal white adipose tissue (gWAT) compared to subcutaneous inguinal white adipose tissue (iWAT) or interscapular brown adipose tissue (BAT) (Figure 1A). In contrast, male mice had a similar pattern of Lum expression across all fats. When mice were housed on HFD for 10 weeks, Lum expression was increased in female gWAT but not iWAT (Figure 1B). Interestingly, we observed that after as little as 1 week of HFD in young female mice Lum mRNA and protein levels had increased (Figure 1C,D). Furthermore, in obese patients, the visceral (oWAT) depot had significantly higher Lum mRNA compared to the subcutaneous depot (pWAT) (Figure 1E).

**Figure S1:**
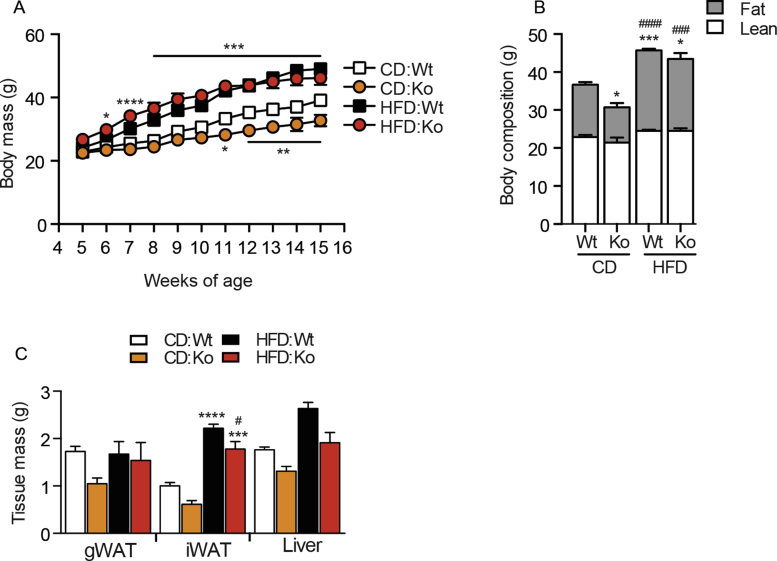


**Figure S2:**
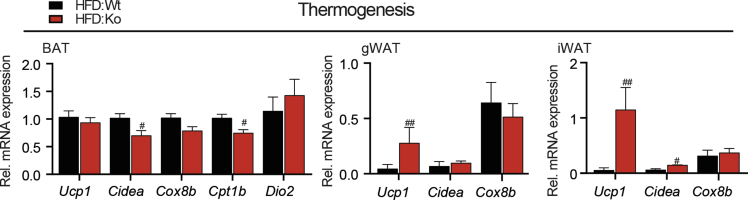


Together these results suggested the intriguing possibility of Lum involvement in adipose tissue growth especially under dietary challenge. We speculated that increased Lum was contributing to deleterious outcomes coupled with significant fat accumulation under HFD conditions [15], [34]. To address this hypothesis, we began studies with *Lum* knockout (*Lum-*Ko) mice previously developed in the Kao lab [22]. Surprisingly, female *Lum-*Ko mice showed greater weight gain during 10 weeks of HFD compared to female wild-type (Wt) mice (Figure 1F), and this was due to increased total body fat mass as measured by EchoMRI (Figure 1G). Although *Lum*-Ko males on CD became leaner compared to Wt, there was no difference in fat accumulation between genotypes on HFD (Figure S1A,B). To explain the increased fat mass in *Lum-*Ko females on HFD, we assessed energy balance in *Lum-*Ko and Wt female mice on HFD by indirect calorimetry. Food intake displayed a normal diurnal pattern in both genotypes with a 2–3% decrease in RER in *Lum*-Ko mice (Figure 1H). After using ANCOVA to adjust for differences in body mass, oxygen consumption (VO_2_) and total energy expenditure (TEE) were not different between genotypes during the dark or light phase (Figure 1H). The gene expression pattern of thermogenic markers in BAT, gWAT and iWAT did not indicate a systemic reduction in thermogenic activation (Figure S2). Notably, ambulatory activity was decreased compared to Wt, implying that the increased fat accumulation in HFD *Lum-*Ko mice may be due, in part, to decreased movement (Figure 1H).

### 3.2 High fat diet promotes increased inflammation in *Lum-*Ko females

**Figure S3:**
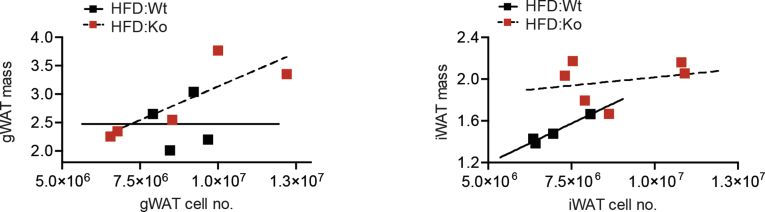


In agreement with the body composition data, *Lum-*Ko female mice on HFD had moderately increased gWAT and iWAT mass compared to Wt (Figure 1I). Male mice on HFD had no change in gWAT, but increased iWAT mass compared to CD fed mice (Figure S1C). To address the adipose tissue cellularity in *Lum-*Ko and Wt on HFD we performed immunofluorescence for the membrane protein Caveolin-1 (Cav-1). Surprisingly, despite the significant increase in adipose growth, adipocyte areas were not significantly different between *Lum-*Ko and Wt females (Figure 1J). A trend of higher total adipocyte number in *Lum*-Ko iWAT was observed and overall, the correlation between depot mass and adipocyte number did not reveal compromised hyperplastic growth in *Lum*-Ko WAT depots (Figure 1K and S3). Next, we studied the cells responsible for new adipocyte formation. Sorted cell populations from the stromal vascular fraction and mature adipocytes from mice fed CD or HFD for 1 week were analyzed for Lum mRNA expression. The adipose progenitor fraction identified previously as Lin-CD34+Sca1+ [35] had markedly higher expression of *Lum*, which was further induced upon HFD (Figure 1L). Likewise, stromal vascular cells isolated from human WAT displayed significantly higher *LUM* mRNA levels compared to mature adipocytes and whole WAT (Figure 1M). Within the HFD cohorts Wt and knockout female mice had a similar number of Lin-CD34+Sca1+ cells (Figure 1N). However, after 7 days of ex vivo differentiation, *Lum-*Ko Sca1+ cells had a slightly diminished capacity to accumulate lipids, as measured by Oil Red O stain (Figure 1O). Together these data suggested no major effect on WAT expandability in *Lum-*Ko mice.

**Figure 2:**
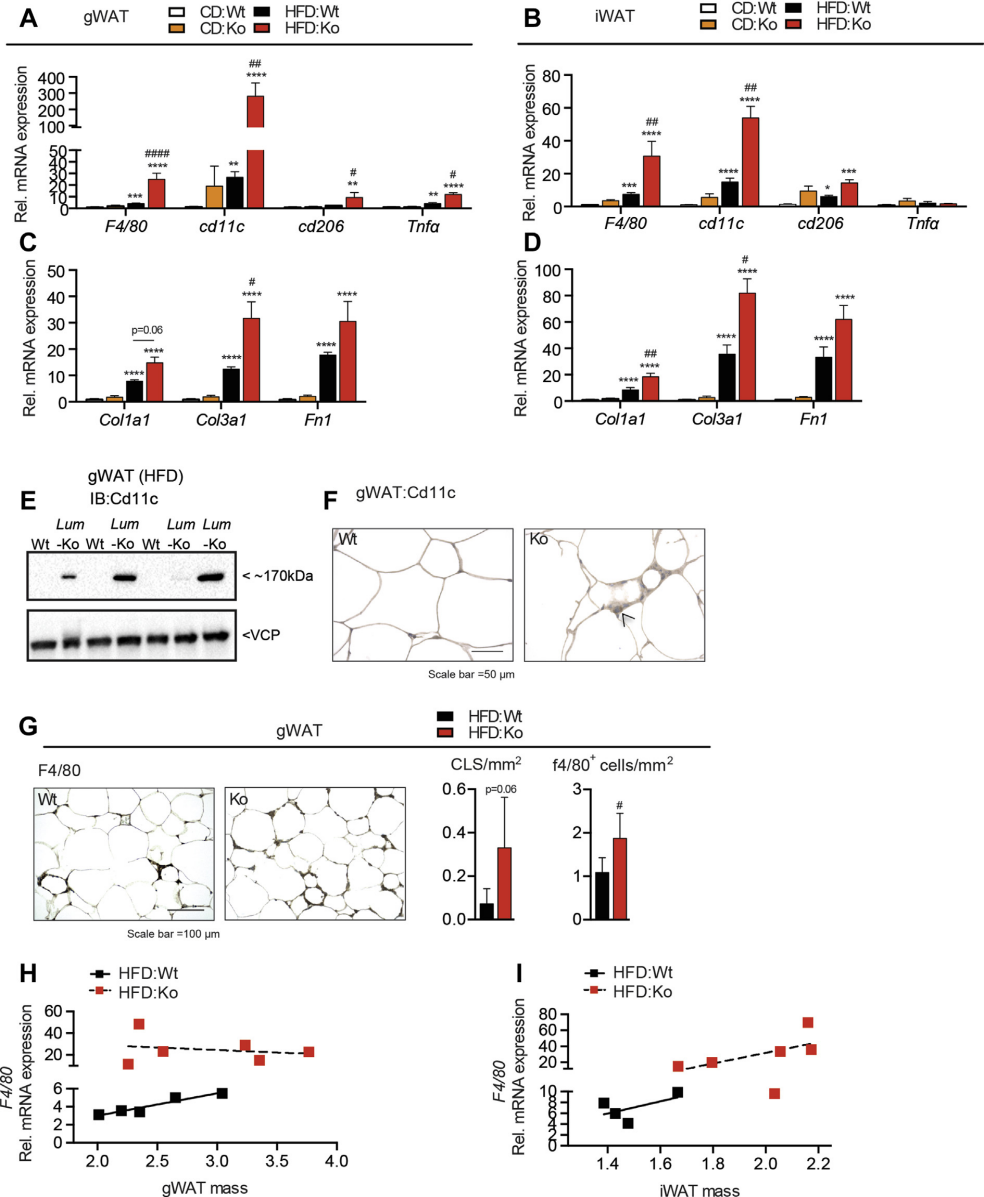
***Lum-*Ko female mice have increased adipose tissue inflammation**. Markers of inflammation and fibrosis in gWAT (A and C) and iWAT (B and D). Western blot (E) and IHC (F) for Cd11c in gWAT for *Lum-*Ko and Wt on HFD. Representative F4/80 immunohistochemistry images, CLS counts and F4/80-positive cell counts in gWAT (G). Correlations between *F4/*80 mRNA and gWAT (H) or iWAT (I) mass. Data are mean ± SEM. n = 3–6 per group. Statistical analysis was performed using two-way ANOVA with posthoc Tukey's test for all panels except G where t-Test was used; p-values as described in Figure 1; Cd11c positive cell (black arrow head); crown-like structures (CLS).

**Figure S4:**
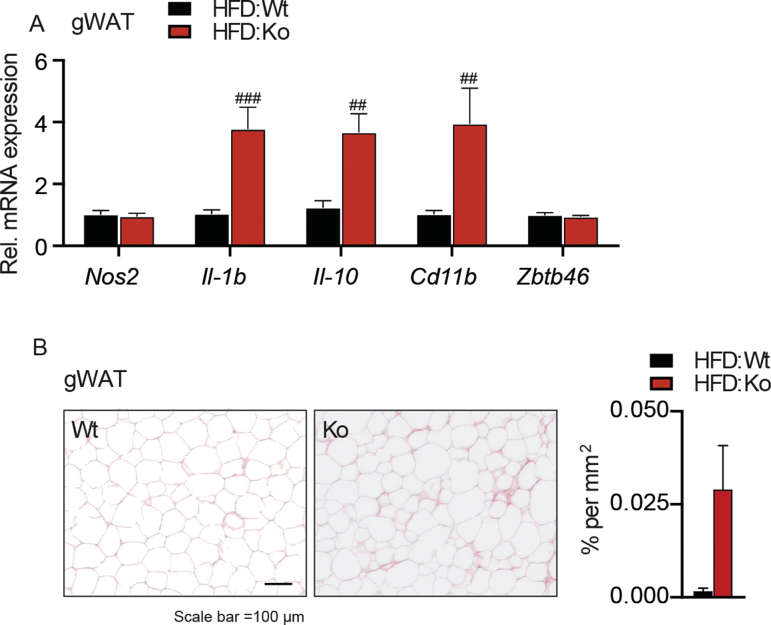


To further understand the qualitative, depot-specific effects of *Lum* deficiency during obesity development, we measured markers of inflammation in gWAT and iWAT. mRNA expression of general (*F4/80*, *Cd1*1b), proinflammatory (*Cd11c*, *Tnfa*, *Il1b*) and M2-type (*Cd206*, *Il10*) macrophage markers but not of the dendritic cell marker *Zbtb46* was increased in *Lum*-Ko mice (Figure 2A,B and S4A). Likewise, markers of fibrotic progression *Col1a1*, *Col3a1*, and *Fn1* were highly increased in HFD, *Lum-*Ko females, both in gWAT and iWAT, which was reflected in a trend of increased Picrosirius red collagen staining (Figure 2C,D, S4B). As reported previously [19], [36], mRNA of the proinflammatory *Cd11c* marker was induced by HFD but showed a marked upregulation in *Lum*-Ko gWAT under HFD. This resulted in robust detection of Cd11c protein in *Lum*-Ko gWAT which appeared to be localized in areas of immune infiltration (Figure 2A,E,F). Indeed, crown-like structures (CLS) and F4/80-positive cells were increased in the gWAT of *Lum-*Ko female mice (Figure 2G). We asked whether the increased inflammation was related to differential depot expansion under HFD. We saw a clear positive relationship between iWAT size and *F4/*80 mRNA expression, however, increased inflammation in *Lum-*Ko gWAT was independent of depot size (Figure 2H,I).

### 3.3 *Lum-*Ko females become insulin-resistant on high fat diet

**Figure 3:**
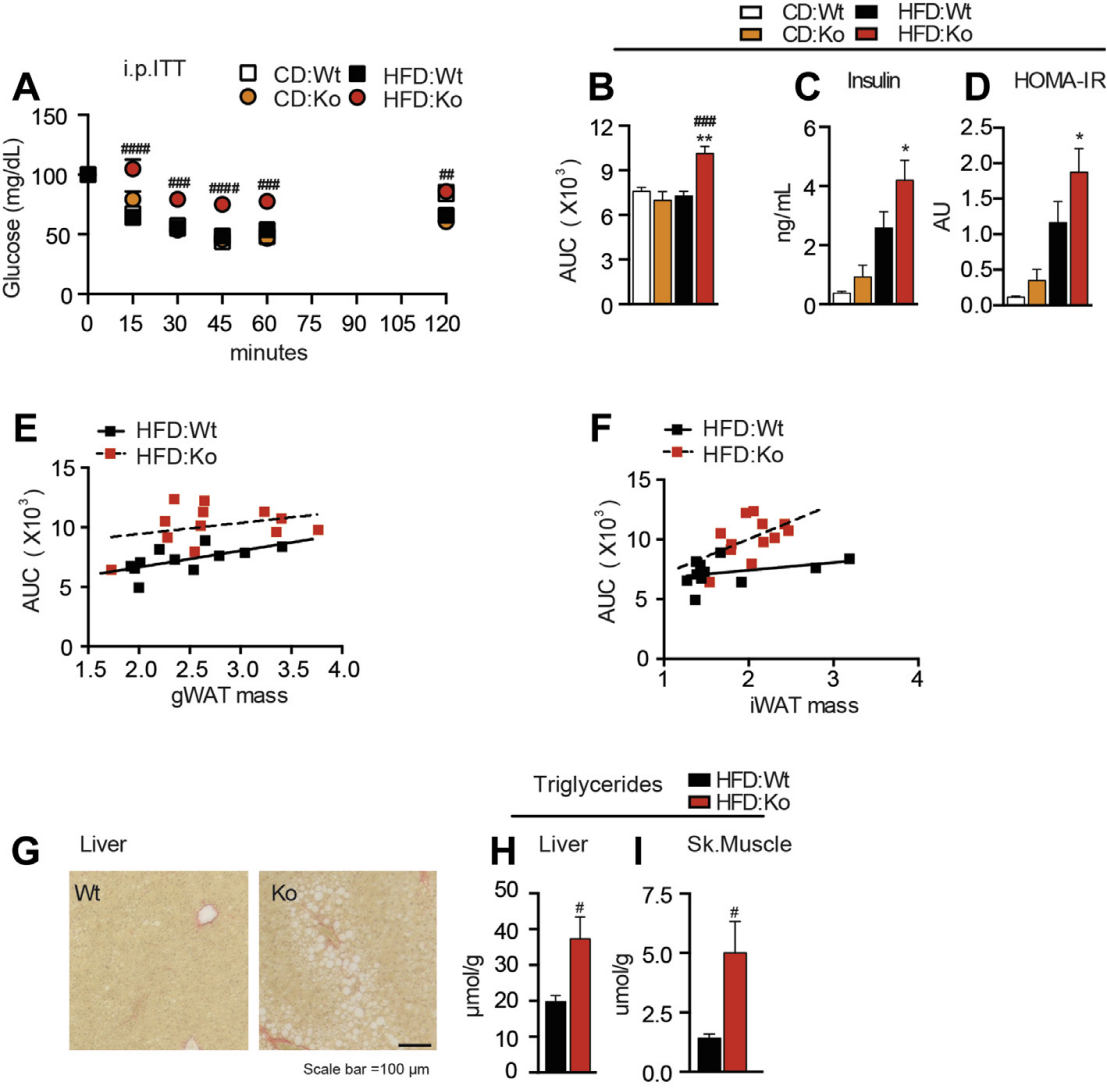
***Lum-*Ko female mice display hallmarks of the metabolic syndrome**. Intraperitoneal insulin tolerance test (i.p. ITT) performed at week 10 of diet (A) and AUC (B). Insulin levels (C) and HOMA-IR (D) from 6 h fasted serum at terminal collection. Correlation between i.p. ITT AUC and gWAT (E) or iWAT (F) mass. Liver lipids, representative images (G) and triglyceride concentration from liver (H) and skeletal muscle (I) were measured at week 10 of diet. Data are mean ± SEM. n = 3–12 per group. Statistical analysis was performed using two-way ANOVA with RM and posthoc Sidak test for A, two-way ANOVA with posthoc Tukey's test for B-D, and t-Test for H and I; p-values as described in Figure 1.

**Figure S5:**
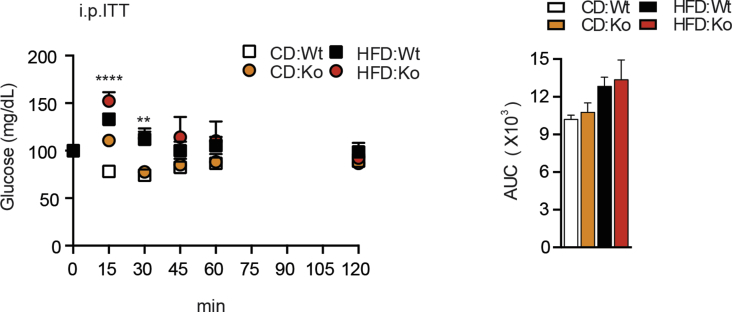


**Figure S6:**
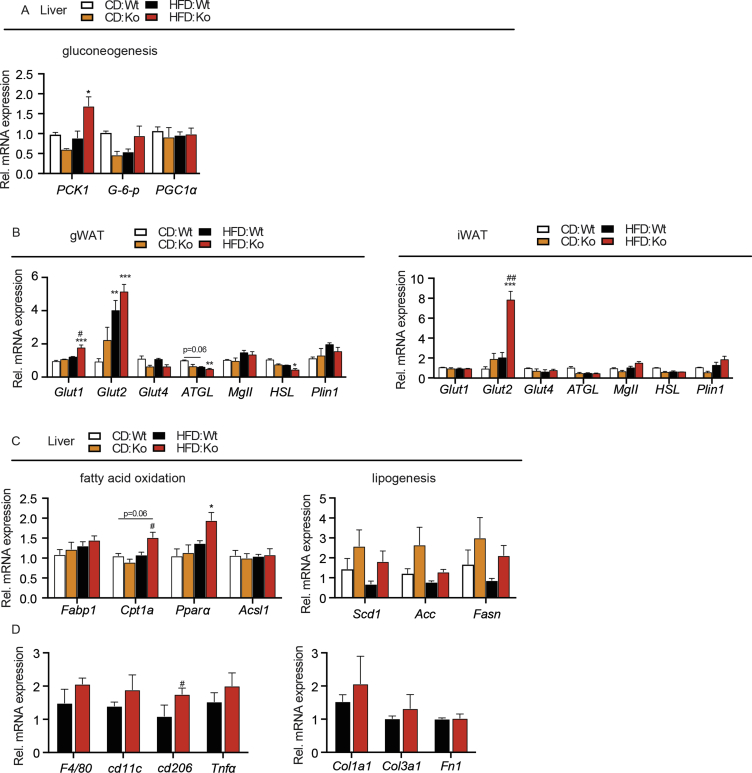


In conjunction with changes in adipose quantity, quality, and inflammation status we observed hallmarks of the metabolic syndrome in *Lum-*Ko female mice on HFD. Insulin tolerance tests revealed that female *Lum-*Ko mice on HFD became insulin-resistant as compared to Wt (Figure 3A,B). In contrast, *Lum-*Ko male mice on HFD had a similar response to insulin as Wt (Figure S5). The impaired insulin response in *Lum*-Ko females was consistent with elevated fasting insulin and HOMA-IR values (Figure 3C,D). Increased insulin resistance corresponded with a modest increase in liver gluconeogenic *Pck1* expression (Figure S6A). Intriguingly, when comparing mice with similar gWAT or iWAT mass between genotypes, *Lum*-Ko mice showed a poorer response in the ITT (Figure 3E,F). However, expression of neither the glucose transporters or key lipolysis genes could explain the impact of adipose tissue on systemic glucose homeostasis in Lum-Ko mice (Figure S6B). The liver and skeletal muscle of *Lum-*Ko mice had increased lipid droplets and triglyceride content (Figure 3G,I), in the absence of increased liver inflammation or pathogenic changes in markers for lipid handling in the liver (Figure S6C,D). Together, these data suggest a role of Lum in adipose tissue and systemic lipid and glucose homeostasis under caloric excess and further imply a connection with the development of key factors of the metabolic syndrome in females.

### 3.4 Lum over-expression improves glucose homeostasis

**Figure 4:**
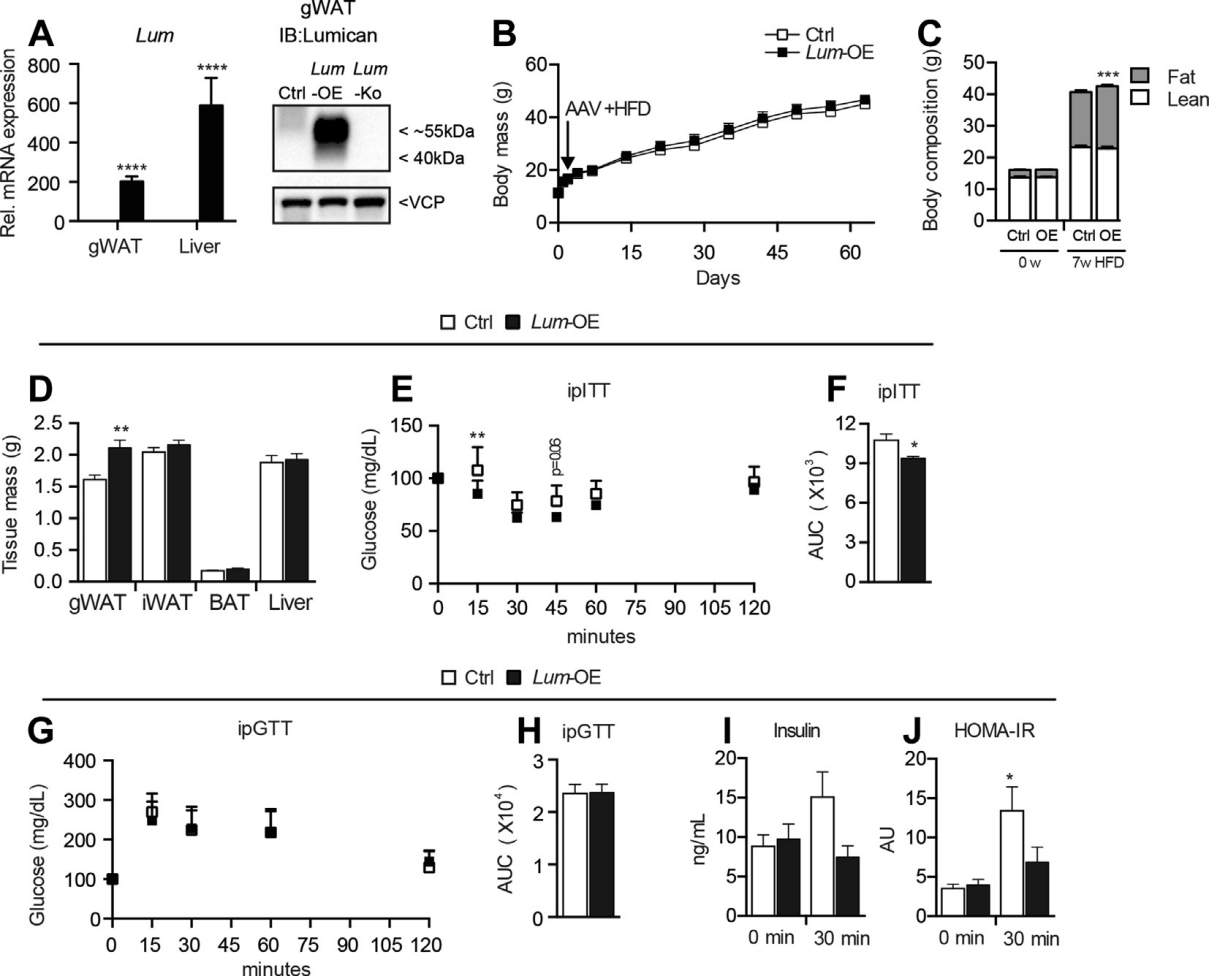
**Over-expression of Lum increases insulin sensitivity**. Male Wt mice at 5 weeks of age were injected i.p. with 2 × 10^11^ vector particles (v.p.) of AAV-GFP (Ctrl) or AAV-Lum (*Lum-*OE), then fed HFD *ad libitum* for 8 weeks. *Lum* mRNA levels at week 8 in liver and mRNA and protein in gWAT (A). Body mass (B), body composition as measured by EchoMRI (C), and tissue masses (D). i.p. ITT (E) and AUC (F). Intraperitoneal glucose tolerance test (i.p. GTT) (G) and AUC (H). Insulin levels (I) and HOMA-IR (J) from 6 h fasted mice before injection (0 min) and 30 min post-glucose i.p. administration. Data are mean ± SD in B, E, and G, all others are mean ± SEM. n = 7–8 per group. Statistical analysis was performed using *t*-Test for A, D, F, and H, for I and J paired t-Test was performed and B, C, E, and G where two-way ANOVA with RM and posthoc Sidak test was performed; *p < 0.05, **p < 0.01, ***p < 0.001, ****p < 0.0001 vs. Ctrl. (for J, *p < 0.05 vs 0 min Ctrl).

**Figure S7:**
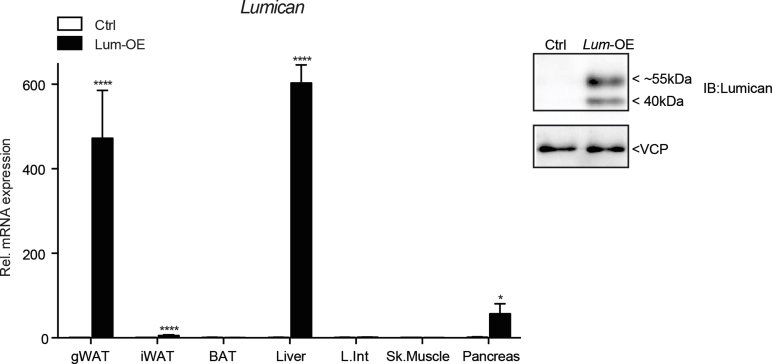


To gain further insight into the role of Lumican in diet-induced obesity and insulin resistance without the influence of any developmental and behavioral functions of Lum, we over-expressed Lum in key visceral organs through a recombinant adeno-associated virus delivered intraperitoneally. To ensure over-expression in the stromal cells, which are normally contributing most of the tissue Lum expression and ECM (Figure 1L), we used a ubiquitously expressed promoter [37]. Male mice were used, which are more prone to insulin resistance and express lower levels of Lum in gWAT (Figure 1A) [38]. Three weeks after infection, we detected a robust and significant increase in gWAT *Lum* mRNA and protein and liver *Lum* mRNA levels in *Lum-*OE mice without widespread systemic over-expression (Figure S7). Following 8 weeks on HFD, Lum mRNA and protein was still highly elevated in gWAT and liver of *Lum-*OE mice (Figure 4A). In the weeks after AAV injection, all mice consistently gained weight, but on week 7 of HFD, a small but significant increase in fat mass was detectable in *Lum-*OE mice, which was partly due to higher gWAT mass (Figure 4B–D). However, triglyceride levels in the liver and skeletal muscle were similar between Ctrl and *Lum*-OE mice (Figure S8A,B). Surprisingly, although *Lum-*OE mice had more fat, they were more insulin-sensitive compared to controls as measured by i.p. ITT (Figure 4E,F). While i.p. GTT did not show any differences in glucose handling, the reduced insulin levels and HOMA-IR at 30 min during GTT indicated improved peripheral insulin sensitivity (Figure 4G–J) with no changes in liver metabolic markers (Figure S8C). Together, these data support a role of Lum in glucose homeostasis and insulin sensitivity in a diet-dependent manner.

## 4 Discussion

The results from this study suggest a role of the ECM protein Lum in the development of diet-induced obesity and insulin resistance. *Lum-*Ko females showed 10–20% increased fat accumulation compared to Wt and this was specific to the HFD treatment. Although the decreased cage activity of the *Lum*-Ko mice represents a plausible explanation for this, energy expenditure was not different between genotypes, which could be due to the weak association of activity with total energy expenditure in mice under the current experimental conditions [4], [39], [40]. The expression of thermogenic marker genes in BAT and WAT did not indicate systemically altered thermogenic activation as an explanation for the positive energy balance in *Lum*-Ko mice. In contrast to our hypothesis, adipose tissue expandability was not compromised in HFD-fed *Lum*-KO mice, in that there was no major genotype effect on adipocyte size and number or the adipogenic capacity of progenitor cells. However, *Lum* inactivation affected adipose tissue quality as determined by the elevated expression of markers of inflammation and ECM/fibrosis. In iWAT, F4/80 expression positively correlated with depot size, possibly reflecting systemic energy balance. Intriguingly though, gWAT, which expressed higher *Lum* levels than iWAT in Wt mice, showed substantially increased F4/80 expression in *Lum*-KO mice independently of depot size.

In accordance with the unhealthy adipose tissue phenotype, *Lum*-KO females were insulin resistant. Although this could be related to altered energy balance, insulin tolerance was worse in *Lum*-KO mice compared to Wt mice with similar fat mass based on correlation analyses. In addition, the improved insulin sensitivity upon *Lum* overexpression occurred without a reduction in fat mass. The contribution of altered adipose tissue quality to the systemic glucose homeostasis phenotypes cannot be proven in the absence of tissue specific perturbation models. Regarding the contribution of other organs it is noteworthy that (A) the liver of *Lum*-KO mice did not display increased inflammatory and fibrotic marker expression, (B) liver fat accumulation was not altered by *Lum* overexpression, and (C) skeletal muscle was not efficiently infected in the AAV-*Lum* overexpression model and thus unlike to contribute.

The effects of *Lum* inactivation on HFD-induced fat accumulation and insulin sensitivity were restricted to female mice. This could be related to the differential pattern of *Lum* adipose tissue mRNA expression we observed between sexes or a lower requirement for *Lum* function in males. Nevertheless, males were responsive to *Lum* overexpression. Interestingly, ovariectomy or estrogen receptor-β inactivation have been shown to result in reduced Lum expression and altered collagen organization in mouse skin, implying a general role of estrogen in the regulation of Lum expression and ECM structure [41], [42]. Future investigations will elucidate the role of Lum in sex-specific ECM and metabolic regulation.

Lum expression was higher in intra-abdominal than in subcutaneous fat depots in female mice and obese humans and was rapidly upregulated in mouse gWAT by HFD feeding. Thus, it is conceivable that Lum is part of an adaptive program for the reorganization of the ECM in response to nutrient overload and the concomitant inflammatory stress in adipose tissue, in analogy to its role in wound healing [9], [43]. The functional and molecular links between Lum, ECM structure, inflammation and adipose tissue metabolism remain to be determined.

## 5 Conclusion

Although increased serum Lum levels have been associated with increased collagen and fibrosis in patients with NAFLD and NASH [16], our data suggest that exploring the tissue-specific role of Lum in the context of overnutrition may provide new insights into the complex interactions between the ECM, inflammation, and the metabolic syndrome.

## Figures and Tables

**Figure S8:**